# Education Research: Simulation-Based Interventions in Acute Stroke Care From Symptom Onset to Acute Treatment: A Scoping Review

**DOI:** 10.1212/NE9.0000000000200263

**Published:** 2025-10-29

**Authors:** Soffien Chadli Ajmi, Thomas Bailey Tysland, Bastian Volbers

**Affiliations:** 1 Department of Neurology, Stavanger University Hospital, Norway; and; 2 Department of Neurology, Inselspital, Bern University Hospital, Switzerland.

## Abstract

**Background and Objectives:**

Simulation-based interventions serve several purposes in acute stroke care. The diversity of reported objectives, techniques, and outcomes makes it difficult to assess effectiveness and derive guideline recommendations. This scoping review aims to map existing research to identify knowledge gaps and suggest future directions.

**Methods:**

This review was conducted in accordance with the Joanna Briggs Institute guidelines for scoping reviews. Empirical research on simulation training targeting health care professionals in acute stroke care from symptom onset to acute treatment was included. We searched MEDLINE, Embase, Education Resources Information Center, ClinicalTrials.gov, and International Clinical Trials Registry Platform. Two independent blinded reviewers performed selection and data extraction using Rayyan.ai, with additional verification by Consensus AI. We performed descriptive analyses and visualized key characteristics.

**Results:**

Fifty-nine studies (95% published between 2015 and 2025) were included. Objectives were categorized as system improvement (31%), educational (31%), technical skills (20%), research (14%), and diagnostic (5%), each with a range of reported techniques (role-playing, task trainer, 3D printing, and virtual reality). There is a paucity of data on the application of simulation for competency assessment, continuing education, specific scenarios such as intracerebral hemorrhage, its application in the prehospital setting, and remote delivery. Several studies lacked reporting of patient-related outcomes (52% of 48 eligible). Most reported outcomes were short-term effects of a single simulation session (85%), limiting the assessment of long-term impact. Inadequate study design and reporting, combined with insufficient details on simulation conduct, particularly debriefing, psychological safety, and resource utilization, limit the inferences that can be drawn from the included studies.

**Discussion:**

We reported a diverse range of objectives, techniques, and outcomes of simulation-based interventions in acute stroke care. There is limited evidence on the translation of simulation training effects to clinical practice and their long-term sustainability. Further research should strengthen the link between simulation interventions, their conduct, patient-related outcomes, and sustainability. In addition, exploring underused applications may help broaden the role of simulation in acute stroke care. The scientific assessment of simulation intervention effectiveness requires standardized reporting, long-term outcome measurement, and study designs that allow for causal inference.

## Introduction

Simulation-based interventions in health care are diverse and can serve a wide range of purposes. In Gaba's seminal paper from 2005, the diverse applications of simulation in health care are categorized along 11 dimensions (e.g., aims and purposes and unit of participation), with each dimension further divided into 5 categories, illustrating the broad application space.^1^ This diversity is reflected in acute stroke, from team training to thrombectomy skill development.^2,3^ Despite its potential to improve stroke care, there are no specific recommendations for simulation-based interventions in the American Heart Association's acute stroke guidelines.^4^ By contrast, the guidelines for Cardiopulmonary Resuscitation and Emergency Cardiovascular Care include recommendations for training based largely on systematic and scoping reviews.^5^

A European Stroke Organization Simulation Committee article on standards of methodology for simulation in acute stroke provides examples of typical *objectives* (e.g., improving technical skills, workflow optimization, and testing protocols), *techniques* (e.g., role-playing, procedural simulation, and virtual reality), and *outcomes* (e.g., satisfaction of participants, knowledge, skills, attitudes, or patient-related outcomes) reported in simulation-based interventions.^6^ Existing literature reviews have focused on aspects such as methodology, utility, overall effectiveness, and potential.^7-9^ Although helpful for guiding conduct and research of simulation-based interventions, the heterogeneity in *objectives*, *techniques*, and *outcomes* makes it difficult to assess the relative or overall effectiveness, even in nonscoping systematic reviews. Simulation in acute stroke remains under-reviewed, with a paucity of studies systematically mapping characteristics of simulation to clarify the observed heterogeneity. This highlights the need for a scoping review to inform future research.

The aim of this scoping review was to map *objectives*, *techniques*, and *outcomes* of simulation-based interventions for health care professionals in acute stroke to identify knowledge gaps and inform future areas of research. This may serve as a foundation for more targeted systematic reviews and future guideline development.

## Methods

### Standard Protocol Approvals, Registrations, and Participant Consents

A preliminary search of PROSPERO, the Cochrane Database of Systematic Reviews, Joanna Briggs Institute Evidence Synthesis, ClinicalTrials.gov, and International Clinical Trials Registry Platform (ICTRP) identified no ongoing systematic reviews. This scoping review was performed according to the Joanna Briggs Institute updated methodological guidance (Table 1).^10^ The detailed review protocol was published on Figshare on October 22, 2023.^11^ Key protocol features and amendments are reported here.

**Table 1:** Frameworks Used for Reporting and Assessment of Knowledge Gaps in This Review

Frameworks	Description	Use in this review
Joanna Briggs Institute updated methodological guidance and PRISMA-ScR^11,12^	Guidance for the conduct and reporting of a scoping review, flow diagram for reporting the selection of sources of evidence	Framework for conducting and reporting this review
Peer Review of Electronic Search Strategies: 2015 Guideline Statement^13^	Guidance to improve the peer review of electronic literature search strategies	The electronic search strategy was peer-reviewed using this guideline statement
Reporting guidelines for health care simulation research: extensions to the CONSORT and STROBE statements^14^	Reporting guidelines for simulation-based research	Includes key elements to report for simulation-based research. These items were used to develop the variables used in the charting table of this review
Kirkpatrick model for training evaluation^15^	Classifies reported outcomes of training interventions on a 4-level scale ranging from participant reactions, learning, behavioral change, and results	Outcomes of simulation-based interventions were classified according to the highest reported Kirkpatrick level to provide an indication of gaps in the translation from simulated (levels 1 and 2) to clinical practice (levels 3 and 4)
Gabas' framework for simulation application^1^	Describes the theoretical application space of simulation interventions along 11 dimensions and 5 categories per dimension	To derive gaps in the application of simulation, the actual application derived from this review was compared with the theoretical application described in Gabas' framework

Abbreviations: CONSORT = Consolidated Standards of Reporting Trials; PRISMA-ScR = Preferred Reporting Items for Systematic Reviews and Meta-analyses; STROBE = Strengthening the Reporting of Observational Studies in Epidemiology.

This study did not require ethical approval, and because there were no individual participant data, no consents were required.

### Eligibility Criteria

This review includes empirical research on simulation-based interventions targeting health care professionals involved in the treatment of acute stroke patients, from symptom onset through the administration of acute therapy. This excludes educational interventions aimed at medical students, non–health care professionals, or patients. Simulation-based interventions were defined as preplanned interventions that replace or amplify real experiences with guided ones.^16^ This excludes e-learning courses and traditional lectures. All sources reporting characteristics of the simulation-based intervention and at least 1 outcome were considered for inclusion, even if part of a broader course. Reviews not reporting empirical research, conference abstracts, unpublished materials, texts, and opinion papers were not considered for inclusion.

During inclusion criteria testing, eligibility criteria from the prepublished protocol were refined for clarity: studies on carotid stenting or diagnostic angiography without a thrombectomy scenario and those focused on traumatic intracerebral hemorrhage (ICH) were excluded.

### Search Strategy

We searched the following databases for published studies (the date of the most recent search in parenthesis): MEDLINE (May 6, 2025), Embase (May 6, 2025), Education Resources Information Center (ERIC) (May 6, 2025), ClinicalTrials.gov (May 6, 2025), and ICTRP (May 6, 2025). We developed a full search strategy for MEDLINE and Embase (eAppendix 1) based on index terms and keywords extracted from a preliminary review. The search strategy was then adapted to ERIC, ClinicalTrials.gov, and ICTRP and peer reviewed according to the Peer Review of Electronic Search Strategies guideline statement (Table 1).^17^ The reference list of all included sources of evidence was screened for additional studies using Citation Gecko. No restrictions were applied to date or language.

### Selection of Sources

**Figure 1:**
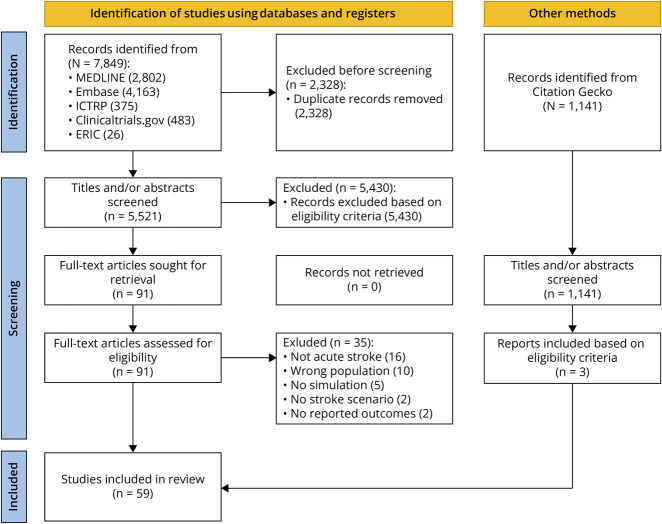
Preferred Reporting Items for Systematic Reviews and Meta-Analyses Flowchart Depicting the Literature Research and Inclusion Process ERIC = Education Resources Information Center; ICTRP = International Clinical Trials Registry Platform.

After deduplication, we exported all identified articles to Rayyan.ai.^13^ Rayyan.ai is a web-based tool for blinded screening, tagging, and conflict resolution. The review was performed in 2 steps by 2 independent and blinded reviewers. Any disagreement between the reviewers at each stage of the selection process was resolved through discussion. Search and inclusion results are presented in a Preferred Reporting Items for Systematic Reviews and Meta-analyses extension for scoping review flow diagram (Figure 1).^18^

### Data Charting Process and Items

One reviewer extracted data; a second verified it (eAppendix 2). We also used Consensus AI to verify charted data. This tool supports data verification using large language models and provided a third level of verification. The prompts used are provided in supplemental material (eAppendix 3). *Objectives*, *techniques*, and *outcomes* of the studied simulation interventions were the main outcome measures. To identify other characteristics, we used an established framework for reporting simulation-based research as a guide (Table 1).^12^ Authors were contacted if there were any missing data related to the main outcome measures.

### Synthesis of Results

#### Data Analysis and Presentation

The objectives and techniques of all included studies were categorized. We used published frameworks and suggestions for objectives of simulation-based interventions in acute stroke as guidance.^1,9,14^ Barring studies in which simulation was used for research or diagnostic purposes, key outcomes were categorized according to Kirkpatrick levels to highlight the degree of translation of knowledge from simulation to clinical practice (Table 1).^19^ We did not appraise methodological quality or risk of bias of included articles. We visualized included data in an evidence map to provide a rough estimation of typical objectives and techniques for which there is a gap of evidence of translation to clinical practice. Simulation use in stroke (application space) was compared with Gaba's broader application model.^1^

### Data Availability

All data supporting the findings of this scoping review are published in full within the supplementary files. No individual participant data were used, and no additional data sets are available.

## Results

### Selection and Study Characteristics

**Figure 2:**
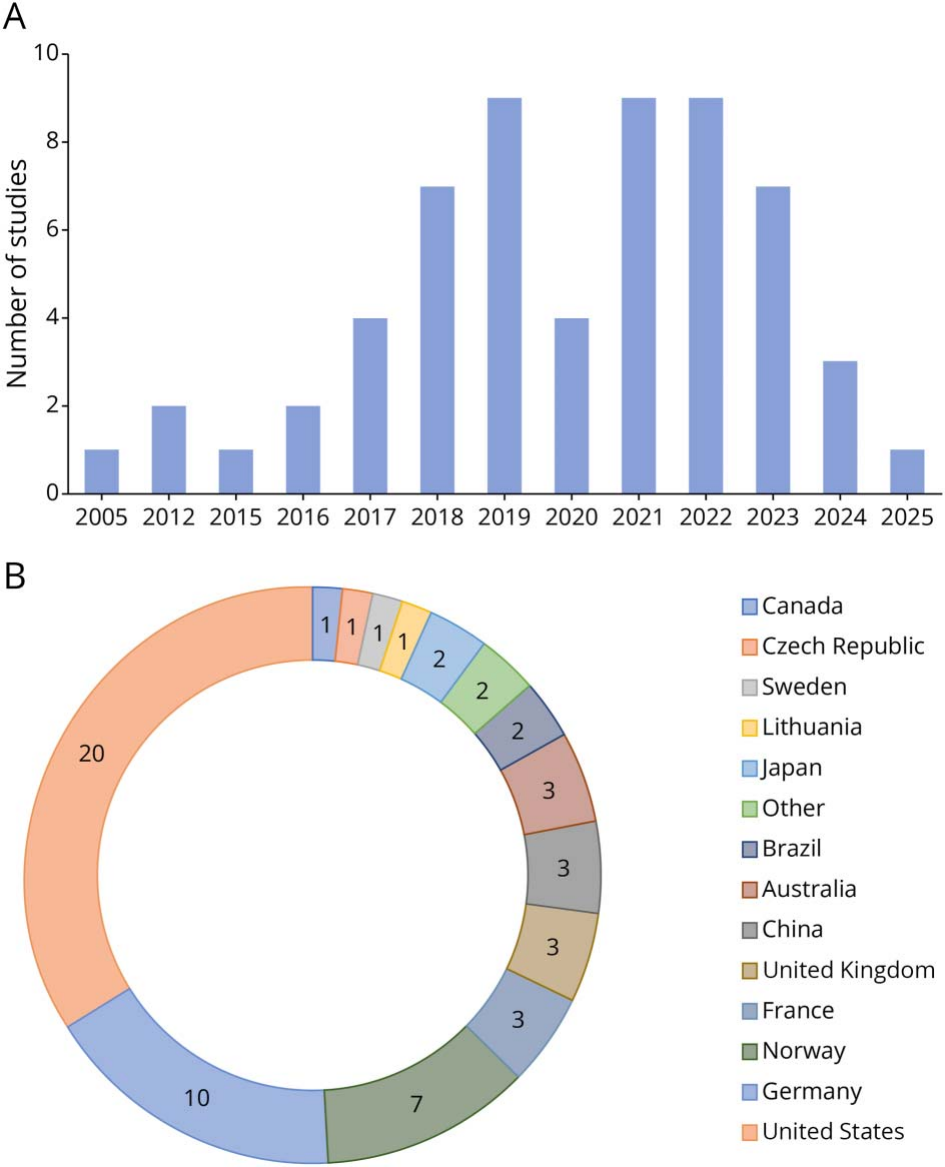
Year of Publication (Top) and Country Where the Simulation Was Conducted (Bottom)

The literature search resulted in 5,521 inclusions after deduplication. After screening, 59 articles were included in this review (Figure 1). Most of the included studies were from the United States (n = 20), followed by Germany (n = 10) and Norway (n = 7). All studies except 3 were published from 2015 until 2025 (eTable 1, Figure 2).

### Characteristics of Sources of Evidence

We categorized the primary *objectives* of the simulation interventions into 5 distinct groups:

1. System improvement: The stated primary aim of simulation is to improve patient outcomes or services (e.g., reducing treatment times for acute reperfusion therapies). This category may also include secondary objectives from categories mentioned further.
2. Educational: Focuses on enhancing knowledge, clinical skills, and human factors, with an emphasis on individual learner development rather than system-level improvements.
3. Technical skills: Aims to improve procedural proficiency in thrombectomies and surgical interventions.
4. Research: Simulation is used as an investigative tool rather than the subject of research, such as validating simulators or assessing aspects of thrombectomy in a simulated environment.
5. Diagnostic: Primarily focused on testing or integrating care pathways.

We also categorized the main simulation *techniques* used across studies into 5 categories:

1. Role-playing: Enacting clinical scenarios to facilitate experiential learning
2. Task trainer: Hands-on practice aimed at developing specific technical skills
3. 3D printing: Using custom anatomical models for skills training
4. Virtual reality: Engaging in immersive, computer-generated simulation environments
5. Other: Techniques that did not fit the above categories. This category includes simulations involving radiology case files, escape rooms, animal models, and alteplase administration.

**Figure 3:**
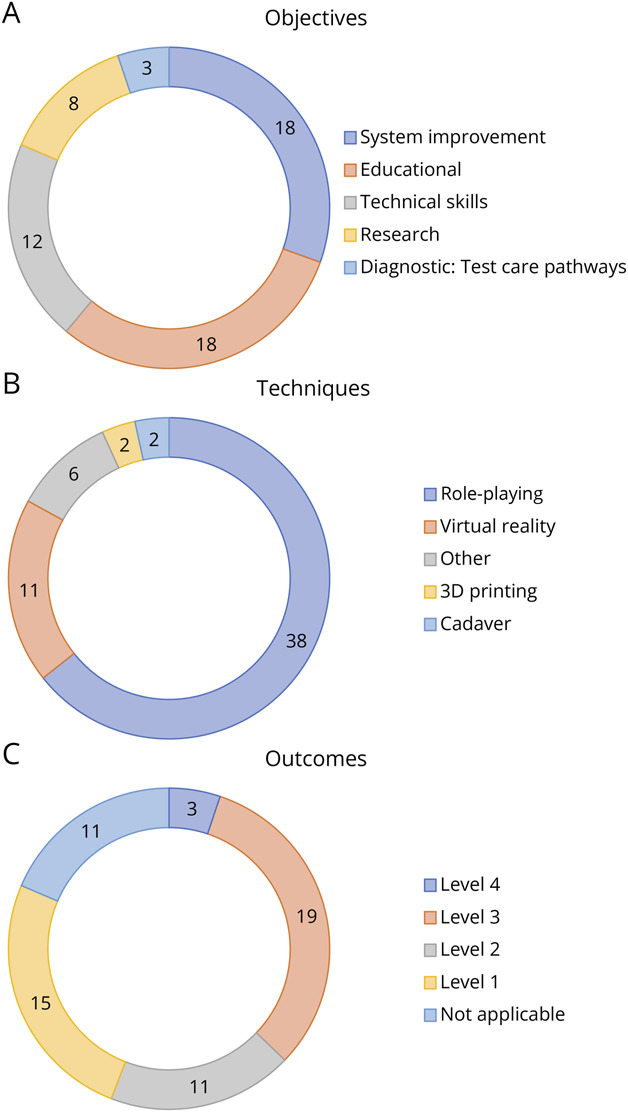
The Number of Included Studies and Reported Objectives^a^ (Top), Techniques (Middle), and Outcomes^b^ (Bottom) ^a^Objectives were classified according to the studies' main objective (e.g., “system improvement” was used for studies with an aim of improving patient outcomes or health services) and the categories for techniques were used to classify the modality. “Other” encompassed radiology cases, escape room, animal models, and alteplase administration. ^b^Kirkpatrick levels from participant reactions (level 1) to patient outcomes (level 4), with higher levels representing translation from simulation to clinical practice.

A summary of the counts of included studies categorized according to *objectives*, *techniques*, and Kirkpatrick level of *outcomes* is presented in Figure 3. All variables from the individual sources of evidence are detailed in eAppendix 2, with a summary table of key variables provided in eTable 1.

### Synthesis of Results

#### System Improvement

##### Objective

Eighteen studies had a primary aim of system improvement (eTable 1). The simulation-based interventions included here often served multiple purposes, such as testing care pathways, enhancing knowledge, and improving communication and team coordination. These interventions were frequently implemented with preplanned concurrent nonsimulation strategies, such as care pathway optimization, to support the goal of system improvement.

##### Technique

All included studies used role-playing and focused on the in-hospital setting, with 13 (72%) being performed in situ. Nine studies (50%) used a mannikin, and the rest a simulated patient. Seventeen simulations (94%) were interdisciplinary team training sessions including parts or all the acute stroke team members as participants.

##### Outcome

**Figure 4:**
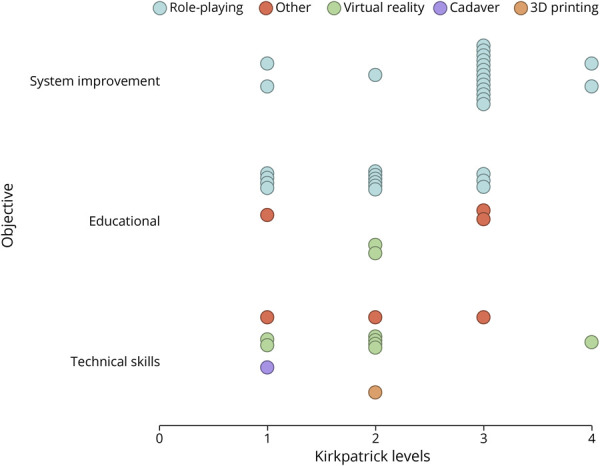
Evidence Map: Simulation-Based Interventions Grouped to Main Objectives^a^ and Kirkpatrick Levels^b^ With Colors Representing the Used Technique^a^ ^a^Objectives were classified according to the main objective of the studies (e.g., “system improvement” was used for studies with an aim of improving patient outcomes or health services) and the categories for techniques were used to classify the modality. “Other” encompassed radiology cases, escape room, animal models, and alteplase administration. ^b^Kirkpatrick levels from participant reactions (level 1) to patient outcomes (level 4), with higher levels representing translation from simulation to clinical practice.

Fifteen studies (83%) within this category reported outcomes at higher Kirkpatrick levels (Figure 4). Most of these further reported outcomes at lower Kirkpatrick levels, such as an increase in provider confidence or knowledge. Of note, 11 of the 15 studies (73%) reporting Kirkpatrick level 3 or 4 had concurrent interventions such as lectures, e-learning courses, or care pathway optimizations, and the reported outcome measures from these studies typically reflect effects of a multicomponent program rather than separating effects of each component. One exception was a multicenter prospective interventional trial. Through studying simulation-naïve vs simulation-experienced teams after care pathway optimization, they assessed the “isolated” effect of simulation training. In this study, the authors found a significant 5-minute door-to-needle time reduction for stroke thrombolysis in favor of simulation-experienced teams.^2^ Two of the included studies reported improved patient outcomes and cost-effectiveness, classifying them as level 4 outcomes. These improvements were observed after the implementation of simulation-based team training as part of a quality improvement project.^15,20^

#### Educational: Knowledge, Skills, and Other Human Factors

##### Objective

Eighteen studies had objectives consistent with the abovementioned definition of educational interventions (eTable 1). Of these, 9 simulation interventions (50%) focused primarily on improving examination skills. The remaining studies focused on increasing confidence, knowledge, or a combination of these. One of these aimed to improve proficiency for junior neurology residents but also used simulation for assessment to compare with senior residents.^21^ Four studies were classified as educational, although the primary aim of the broader multifaceted intervention was system change. In these studies, however, the simulation intervention itself was primarily educational, its effects were measured separately, and we judged that it played a minor role in driving the system change.^22-25^

##### Technique

Thirteen (72%) of these studies used role-playing with mostly simulated patients. The use of simulated patients probably reflects difficulties of using a mannikin to simulate neurologic deficits because 8 of the 10 studies using a simulated patient had, as a primary objective, to improve examination skills. In 1 study, a mannikin was used to improve examination skills, supplemented with videos of real patients with neurologic deficits.^25^ The remaining 5 studies used different techniques. One study assessed the feasibility of a virtual reality–training headset to improve stroke management for neurology residents and found a high acceptance rate and increased self-reported knowledge.^26^ The same technique was also applied for improving proficiency in a telehealth scenario.^27^ Another study used escape rooms (with different puzzles to be solved by the participants using stroke care knowledge) as a technique to achieve increased knowledge for nurse participants.^28^

##### Outcome

Among the reported outcomes in this group, 4 studies (22%) measured outcomes at Kirkpatrick level 3 (Figure 4). In these 4 studies, the reported outcomes were largely driven by concurrent interventions other than simulation or the outcomes were self-reported—meaning that participants claimed behavior changes in real patient care, but no objective patient outcomes were measured.^22,23,29,30^ The remaining 12 studies (75%) reported outcomes at lower Kirkpatrick levels mostly related to confidence, perceived usefulness, and knowledge scores.

#### Technical Skills

##### Objective

Twelve studies aimed to improve technical skills (eTable 1). Of these, 9 studies (75%) specifically sought to improve technical skills in a mechanical thrombectomy scenario while 2 studies (17%) examined a model for training endoscopic ICH surgery and 1 study (8%) aimed to provide hands-on training for alteplase administration. Four (33%) of these studies reported additional objectives that could be categorized as research as they aimed to validate different training modalities.

##### Technique

Seven studies were categorized as virtual reality training, 3 as task training, one as 3D printing (a model that simulates ICH eligible for endoscopic removal), and 1 as a cadaver model. In 6 of the 9 studies on mechanical thrombectomy scenarios, different Mentice VIST simulators were used (defined as virtual reality). The training was organized as either a single-session course or longer program.^31,32^ All included studies assessed a single learner, mostly interventional radiologists or neurosurgeons.

##### Outcomes

All studies reported positive findings at Kirkpatrick levels 1 and 2. These typically included improved confidence or perceived usefulness (level 1) and faster task completion or fewer handling errors measured on a simulator (level 2). Two of the 4 studies that reported additional research-related outcomes found that the Mentice VIST simulator could discern between novices and experts and improve skills in the simulated setting.^3,33^ The other 2 reported that a cadaver model and a 3D-printed model, both for endoscopic ICH removal, were well received and could discern between novices and experts.^34,35^

One study reported a Kirkpatrick level 4 outcome (mortality, early neurologic recovery, and rate of reperfusion) after using the Mentice VIST simulator as part of a comprehensive educational intervention including lectures and proctoring of real cases. There was no pre-post comparison, but outcomes were compared with acceptable quality metrics from guidelines.^33^

#### Research

##### Objective

Eight studies had research as their main objective (eTable 1). Of these, 7 (88%) were validation studies assessing tools such as an eye-tracking device, cadaver models, role-playing simulations for assessment, telehealth implementation, prehospital cognitive aids, and a virtual reality simulator's ability to discern between experts and novices.

##### Technique

Regarding techniques, these varied in accordance with the aim from a 3D-printed phantom, role-playing scenario, and an eye-tracking device. Two studies had a main objective of validating the Mentice VIST simulator through testing its ability to discern between novices and experts.^36,37^ These validated the simulator in a manner similar to the 2 studies reported under “Technical Skills” but are included here because they had a main objective of validation. One study used embalmed human heads with vascular occlusions connected to a flow model inside an Angio suite. The mechanical properties were tested for realism and validated as a method for training with promising capabilities.^38^

##### Outcomes

The outcomes varied widely, reflecting differences in study objectives, all of which were cantered on validation or experimental research. As a result, they could not be classified using the Kirkpatrick framework.

### Diagnostic: Test Care Pathways

Three studies had a main *objective* aiming to test and/or embed care pathways (eTable 1). All 3 used interdisciplinary in-hospital role-playing with simulated patients as a *technique*, in line with their system-focused objectives. *Outcomes* were primarily qualitative (e.g., barriers to interhospital transfer) and included postsimulation confidence surveys. As diagnostic studies, their outcomes could not be classified using the Kirkpatrick framework. However, by targeting latent safety threats and informing implementation, these interventions are well positioned to drive meaningful improvements in patient outcomes.

### Knowledge Gaps and Limitations of Existing Research

The published studies on simulation-based interventions were heterogeneous regarding techniques and outcomes both within and between different groups of objectives (Figures 3 and 4). We identified gaps related to *application*, *outcomes*, *comparative effectiveness*, and *reporting*. These factors are clearly interconnected and influence one another, and although we discuss these issues separately, they cannot be viewed in isolation (Table 2).

**Table 2:** Identified Knowledge Gaps, Limitations, and Suggestions for Future Directions

Knowledge gaps and limitations^a^	Specific gaps	Future directions
Application space: specific applications of simulation with the potential to improve stroke care are understudied or underused	• Objective: assessment • Scenarios: ICH, children, elderly • Context: prehospital • Delivery: remote • Participants: APPs and pharmacists	• Assessment of a wider scope of simulation objectives • Assessment of potential benefit for a larger group of patients with varying characteristics
Outcomes: there is a lack of reports on patient outcomes, return on investment, and sustainability. Very few of the studies reporting patient outcomes are designed for causal inference	• Translation to clinical practice including return on investment • Sustainability • Concurrent non-simulation interventions	• Report patient outcomes and return on investment • Long-term follow-up with assessment of repeated vs single simulations • Design that allows for causal inference
Comparative effectiveness: very few studies compared different techniques or ways of conducting simulation, even within similar objectives	• Comparison of different techniques for similar objectives • Comparison of different ways of conducting simulation	• Assessment of different techniques to achieve the same objectives • Assess and compare different aspects of simulation such as brief, debriefing, and psychological safety
Reporting: reports of simulation interventions are variable making both generalizability and comparing effectiveness of different aspects of simulation difficult	• Standardized outcome measures • Reporting of key aspects of conduct such as briefing, debriefing, psychological safety and resource utilization	• Increase utilization of reporting guidelines to standardize reporting and enable comparison

Abbreviations: APPs = advanced practice providers; ICH = intracerebral hemorrhage.

a The different categories are strongly linked and interact with each other. Gaps and future directions for these issues can, therefore, not be viewed in isolation. For example, comparing effectiveness of certain aspects of simulation presupposes adequate reporting.

**Figure 5:**
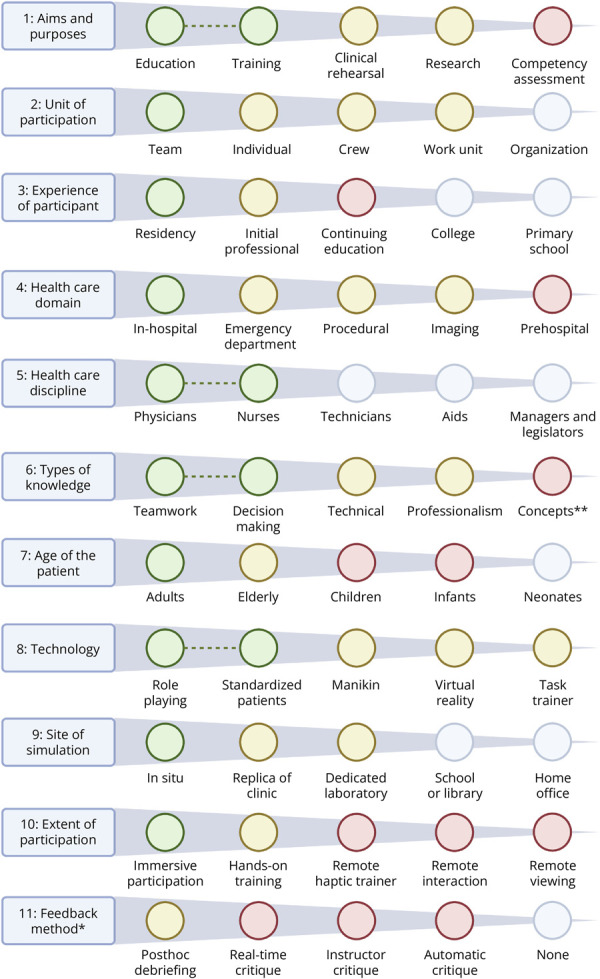
A Schematic of the Application Space of Simulation-Based Interventions According to Dimensions and Categories Suggested by Gaba^1^ The colored circles mark the current applications and gaps of simulation-based interventions in acute stroke. The different categories within each dimension are sorted from right to left in order of increasing occurrence within the included articles. Legend: Green: characteristics reported by 4 or more of the studies in this review and some evidence of translation to clinical practice. Yellow: characteristics reported by 2 or 3 of the included studies or no evidence of translation to clinical practice. Red: potential gaps not fulfilling the aforementioned criteria for red/green. Gray: not relevant because of the eligibility criteria of this review. Green line: characteristics that are typically used within the same intervention. *Difficult to assess because of a lack of sufficient reporting in many included studies. **Several studies reported the education of concepts as part of the simulation intervention; this is, however, marked as a potential gap because there was a relative paucity of intracerebral hemorrhage scenarios.

By comparing the theoretical *application* space of simulation in health care with its actual use in acute stroke care, we identified gaps for competency assessment as an objective, specific scenarios such as ICH, application in the prehospital setting, involvement of different participants, simulations delivered from a remote location, and continuing education (Figure 5). Regarding participants, depending on local emergency workflows, pharmacists or advanced practice providers may also be involved in training, but their inclusion is rarely examined. Two studies offer some insight into simulation applied for continuing education. One found that both novice and experienced physicians improved door-to-needle times after simulation, with novices benefiting most, suggesting value in repeated sessions regardless of experience.^39^ Another study showed that junior residents reached senior-level performance after a brief simulation course but fell short of mastery.^21^ The influence of previous experience and optimal training frequency remains an open question. A large proportion of included studies did not report outcomes on translation to clinical practice, and most reported short-term effects before and after a single simulation intervention. In addition, there was a lack of studies reporting return on investment. Moreover, most of the studies reporting outcomes classified as Kirkpatrick levels 3 and 4 noted concurrent nonsimulation interventions as a potential limitation due to confounding. This is further limited in that ultimately linking objectives and techniques to outcomes requires a study design that allows for causal inference. There is thus an evidence gap regarding both translation and sustainability of isolated simulation-related effects to clinical practice. Because there is an expected clustering between specific techniques and objectives (e.g., virtual reality is frequently used as a technique when the objective is improving technical skills), it is probably not helpful to compare the overall effectiveness of different techniques because these are tailored to the objective (Figure 4). However, even within specific objectives, most included studies did not aim to *compare effectiveness* of different techniques.

An additional barrier to assessing *application space*, *outcomes*, and *comparative effectiveness* is that the *reporting* of simulation conduct including briefing and debriefing techniques, issues related to psychological safety, and resources used, is highly variable and largely lacking. Because these are elements that potentially affect both the intervention and outcomes, their reporting is important for both the interpretation of outcome measures and comparing effectiveness. Moreover, barring studies assessing simulation intervention effects on door-to-needle times, the types of reported outcomes are heterogeneous even within similar objectives, making comparison of effectiveness across studies difficult. Overall, there is thus a knowledge gap in comparing the effectiveness of different simulation techniques for similar objectives, as well as inconsistencies in reporting practices. A summary of knowledge gaps and limitations of the existing literature together with suggestions for future directions is presented in Table 2.

## Discussion

We reported a diverse range of objectives, techniques, and outcomes of simulation-based interventions in acute stroke care. Reported objectives were categorized as system improvement, educational, technical skills, research, and diagnostic. The application space was broad but mostly restricted to improving acute ischemic stroke treatment. Outcomes reported for system improvement were mostly improvements in treatment times during stroke thrombolysis, implying some efficiency in terms of transferability to the clinical setting for this group. For simulation interventions aiming for education or improvement of technical skills, evidence of translation to clinical practice is largely lacking. Methodological issues with study design limit causal inference of reported outcome measures, particularly due to concurrent interventions. Inadequate reporting, particularly regarding the conduct of simulation, further limits generalizability, causal inference, and the possibility of comparing effectiveness of different simulation methods.

The theoretical application space suggested for the broader field of health care simulation by Gaba^1^ is almost infinite. Even within the eligibility criteria for this review, expecting acute stroke simulations to cover all combinations (>48 million unique combinations) is unrealistic and unfruitful. However, we did find a couple of areas in which we probably could advance the application of acute stroke simulation (Figure 5). Important examples include a relative paucity of simulations with an objective of competency assessment. In a notable exception, a prospective observational study found that simulation is a useful method for competency assessment of graduate neurology trainees in the context of acute ischemic stroke management.^40^ Furthermore, there was a paucity of reports using ICH scenarios. With increasing evidence for the importance of timely treatment also in ICH, this represents a potentially underused application.^41^ Potential applications of simulation interventions within the broader field of neurology were also discussed in a thoughtful review focusing on the innovative ways that simulation might be adapted to address current and future educational goals.^9^ We identified several similar gaps. One additional potential application suggested by the referenced review is the evaluation of bias and promotion of equity, which was not an objective of any of the studies we included.

In addition to potential gaps in the application space, there are knowledge gaps in evidence of outcomes and comparative effectiveness. This gap is linked to study design and inadequate reporting, regarding both the conduct of simulation and chosen outcome measures. For most included studies in this review, outcomes reported were typically self-perceived usefulness or confidence. Confidence is a poor surrogate for retained knowledge or improved performance in clinical practice.^42,43^ The studies categorized as system improvement differed in that these more frequently reported outcomes at higher Kirkpatrick levels (Figure 4). This is not surprising because this group had a stated aim of system improvement and thus mostly assessed system-level or patient-level change. Outside of this review, there are several examples of the powerful ways in which simulation can be used for quality improvement.^44^ A recent systematic review with a meta-analysis specifically on simulation training in stroke thrombolysis included all the articles that we classified as system improvement. The authors found a significant mean reduction of 15 minutes in door-to-needle times after simulation training, supporting our statement of potential clinical translation.^45^ While these findings suggest clinical relevance, the most effective way to deliver simulation remains unclear. Comparative effectiveness research is challenging, as simulation should be tailored to specific objectives and contexts. Establishing effectiveness within each objective is a first step. Our objective-based categorization may help define subfields and guide studies comparing simulation with nonsimulation interventions and then different simulation methods targeting the same objective. For example, it remains to be seen whether lower fidelity; less resource-intensive formats; such as table-top exercises; or reduced frequency can achieve similar reductions in door-to-needle time as full-scale in situ simulation. To enable such research, clearer definitions and consistent reporting of context, instructional design, and delivery are needed to support meaningful comparisons.

Are the available data on effectiveness sufficient to justify a guideline recommendation for simulation as a tool for *system improvement*? Regarding objectives, techniques, and reported outcomes, the simulation interventions within the group of system improvement share several characteristics. Most were interdisciplinary team training sessions using simulation in different ways to achieve improvement. There is nonetheless considerable variation, particularly in the use of concurrent interventions (e.g., e-learning, lectures, and care pathway optimizations). This heterogeneity makes it difficult to determine which components are truly effective or redundant.^46^ A further limitation is the lack of research on the sustainability of these interventions. Most studies assess the impact of a single simulation course rather than long-term programs with repeated sessions, making it unclear whether benefits are maintained over time. Similarly, data on return of investment are scarce and especially in resource-intensive settings such as in situ simulations, time and space constraints may limit feasibility. Furthermore, inadequate reporting of key design elements—such as debriefing methods and psychological safety—poses a barrier to informed recommendations.^47,48^ From a guideline perspective, the lack of strong causal links between long-term simulation, patient outcomes, and return on investment, combined with inconsistent reporting, is a barrier to the endorsement of simulation-based training, even for the objective of system improvement.

To provide a foundation for developing specific guidelines on the use of simulation-based interventions in acute stroke care, we suggest the following: We consider it reasonable to focus further on educational aims and system improvement. Within those categories, outcomes reflecting translation to clinical practice should be measured. Those outcomes should be obtained not only directly after the simulation training but also at later time points (e.g., days 90 and 180). The effect of repeated training sessions in comparison with a single session also needs to be evaluated. Regarding methodology, studies should properly adjust for potential confounders such as concomitant interventions or exclude those by design. At best, randomized trial designs could be used including trained and untrained health care groups within the same hospital, and the outcome assessment should be performed in a blinded manner. Reporting should be based on established frameworks and include information about briefing, debriefing, and resources.^6,12^ Finally, aspects such as debriefing and psychological safety should be evaluated and correlated with outcomes.

Although the aforementioned suggestions would strengthen the evidence base and support wider implementation, one could argue that there is enough proof of effectiveness to recommend simulation as a useful tool for a range of purposes in acute stroke. For example, at our center, weekly simulation was used to test and refine treatment protocols, embed changes through rehearsal, and improve teamwork.^15^ While such work is limited by biases and concurrent changes, these adaptations are necessary in complex health care systems and reflect the utility of simulation as a tool for quality improvement.^44^ Rather than viewing heterogeneity and contextual adaptation as limitations, they may signal successful integration into diverse settings. However, the Grading of Recommendations, Assessment, Development and Evaluations framework, which was made for clinical trials and emphasizes causality, often downgrades simulation studies because of risk of bias, indirectness, and heterogeneity. This challenge is also noted in the guidelines for Cardiopulmonary Resuscitation and Emergency Cardiovascular Care, in which the authors state that alternative approaches for appraising educational research will be considered for future evidence reviews.^5^ Finally, the comparator—traditional health care education—has major limitations, little evidence supporting its effectiveness and is unlikely to meet future demands in stroke care.^8,9^ All the abovementioned factors should be considered in a potential guideline recommendation for simulation in acute stroke. Still, robust studies linking simulation to patient outcomes, along with targeted systematic reviews, will be needed to ease the process of providing a guideline recommendation.

This study has several limitations. First, the analysis used to categorize objectives is important regarding the conclusions drawn but is prone to subjectivity as some interventions were difficult to accurately classify. Second, we did not perform any critical appraisal of the included studies. Although critical appraisal is not typically part of a scoping review, according to JBI methodology, it somewhat limits the conclusions made about transferability to clinical practice. Third, the Kirkpatrick framework used to categorize outcome measures has an inherent limitation in that it assumes causality between the intervention and the outcome. Several of the studies included in this review were not designed to assess causality, which, therefore, further limits the conclusions drawn about transferability to clinical practice. Fourth, limiting technical skill simulation to only those that include a thrombectomy scenario might have led to the omission of studies on basic catheterization skills, training in angiography, and elective carotid stenting that might have benefited interventionalists who also treat acute strokes.

System improvement and educational simulations to improve knowledge, clinical skills, and human factors were the most frequently reported objectives for simulation in acute stroke care. Although we saw a diverse range of applications, we discussed the potential to widen the application space. For most objectives, except to some extent system improvement, there was a relative paucity of data on evidence for translation into clinical practice and sustainability. Further research should strengthen the link between simulation interventions, their conduct, patient-related outcomes, and sustainability through study designs that allow for causal inference. In addition, the use of established frameworks to standardize the use, terminology, and reporting of simulation interventions could ease the process of scientific assessment.

## Glossary

ERIC: Education Resources Information Center
ICH: intracerebral hemorrhage
ICTRP: International Clinical Trials Registry Platform
